# Supplementary material for: Post-Mastectomy Breast Reconstruction Disparities: A Systematic Review of Sociodemographic and Economic Barriers
Source: Medicina (Kaunas). 2024 Jul 19;60(7):1169. doi: 10.3390/medicina60071169 (PMC11279340; doi:10.3390/medicina60071169)
Supplement: Supplementary file 1 [file medicina-60-01169-s001.zip › medicina-3060555-supplementary.pdf]

Supplemental Data

Supplemental Table S1.

Study (Year)	Title	Study Design	Data Source	Study Years	Total Study n	Categories
Albornoz (2012)	The influence of sociodemographic factors and hospital characteristics on the method of breast reconstruction, including microsurgery: a U.S. population-based study	Retrospective Cohort	Nationwide Inpatient Sample	2008	15,948	Sociodemographics, Hospital type, Insurance Type, Geography
Alderman (2009)	Racial and ethnic disparities in the use of postmastectomy breast reconstruction: results from a population- based study	Qualitative Cross-Sectional Survey		2005-2007	2,260	Insurance Type
Aliu (2018)	Trends in Medicaid beneficiaries' receipt of breast reconstruction procedures following Pre-Affordable Care Act (ACA) Medicaid expansion in New York State	Retrospective Cohort		1998-2006	133,568	Insurance Type
Beaulieu-Jones (2023)	Impact of Race, Ethnicity, Primary Language, and Insurance on Reconstruction after Mastectomy for Patients with Breast Cancer at an Urban, Academic Safety-Net Hospital	Retrospective Cohort	Single Institution	2009-2014	756	Sociodemographics, Insurance Type, Hospital Type
Boczar (2019)	Influence of Facility Characteristics on Access to Breast Reconstruction: A 12-Year National Cancer Database Analysis	Retrospective Cohort	The National Cancer Database (NCDB)	2004-2015	858,594	Sociodemographics, Income, Education, Hospital Type, Geography
Burke (2022)	Persistent and interdependent: Racial disparities and their mechanisms in postmastectomy	Retrospective Cohort	Multiinstitutional Database	2011-2019	1,554	Sociodemographics, Insurance Type

Study (Year)	Title	Study Design	Data Source	Study Years	Total Study n	Categories
	breast reconstruction					
Butler (2018)	Influence of race, insurance status, and geographic access to plastic surgeons on immediate breast reconstruction rates	Retrospective Cohort	Agency for Healthcare Research and Quality's Health-care Cost and Utilization Project, Area Health Resource File (AHRF), National Cancer Institute's (NCI) Health Service Area (HSA) Multiinstitutional, National	2008-2012	65,246	Sociodemographics, Surgeon, Insurance Type, Geography
Butler (2016)	Racial and age disparities persist in immediate breast reconstruction: an updated analysis	Retrospective Cohort	National Surgery Quality Improvement Program data sets ACS-NSQIP	2005-2011	48,564	Sociodemographics
Connors (2022)	Racial Disparities in Breast Reconstruction at a Comprehensive Cancer Center	Retrospective Cohort	Barnes-Jewish Hospital (BJH) Cancer Registry	2000-2012	4,154	Sociodemographics, Patient Satisfaction
Connors (2016)	Breast reconstruction after mastectomy at a comprehensive cancer center	Retrospective Cohort	Barnes-Jewish Hospital (BJH) Cancer Registry	2000-2012	5421	Sociodemographics, Hospital Type
Corpuz (2023)	Correlating state-specific and national trends in breast reconstruction after Medicaid expansion: A decade-long update on the Affordable Care Act's impact	Retrospective Cohort	Healthcare Cost and Utilization Project State Inpatient Database (HCUP-SID) National Inpatient Sample (HCUPNIS)	2010-2018	109,546	Insurance Type
DeCoster (2020)	Rural-Urban Differences in Breast Reconstruction Utilization Following Oncologic Resection	Retrospective Cohort	Kentucky Cancer Registry (KCR)	2006-2015	10,032	Sociodemographics, Insurance Type, Geography
DeCoster (2019)	Appalachian Status Is a Negative Predictor of Breast Reconstruction Following Breast	Retrospective Cohort	Kentucky Cancer Registry, SEER	2006-2015	12,036	Surgeon, Sociodemographics, Insurance Type, Geography

Study (Year)	Title	Study Design	Data Source	Study Years	Total Study n	Categories
	Cancer Resection					
Enewold (2014)	Breast reconstruction after mastectomy among Department of Defense beneficiaries by race	Retrospective Cohort	DoD's Central Cancer Registry (CCR) and the MHS Data Repository (MDR), the DoD's medical claims database	1998-2007	3682	Sociodemographics, Insurance Type
Epstein (2018)	Racial disparities in postmastectomy breast reconstruction	Retrospective Cohort	American College of Surgeons National Surgical Quality Improvement Program database	2005-2014	92,960	Sociodemographics
Fasano (2022)	Postmastectomy Breast Reconstruction Patterns at an Urban Academic Hospital and the Impact of Surgeon Gender	Retrospective Cohort	Single institution	2018-2021	167	Surgeon, Sociodemographics, Insurance Type
Friedman-Eldar (2022)	Stalled at the intersection: insurance status and disparities in post-mastectomy breast reconstruction	Retrospective Cohort	Multi-hospital institutional database	2011–2019	1,554	Hospital Type, Insurance Type, Sociodemographics
Goldenberg (2022)	Did Medicaid Expansion Mitigate Disparities in Post-mastectomy Reconstruction Rates?	Retrospective Cohort	National Cancer Database	2004-2016	302,791	Insurance Type, Sociodemographics, Geographical
Gooch (2019)	Increases in Postmastectomy Reconstruction in New York State Are Not Related to Changes in State Law	Retrospective Cohort	Statewide Planning and Research Cooperative System database	2005-2015	52,837	Sociodemographics, Insurance Type
Greenberg (2008)	Do variations in provider discussions explain socioeconomic disparities in postmastectomy breast reconstruction?	Retrospective Cohort	NICCQ database	1998	626	Sociodemographics, Communication, Language
Holliday (2017)	Disparities in Rural Breast Cancer Care: Factors Affecting Choice of Breast	Retrospective Cohort	Single Institution, Charleston Area Medical Center	2006-2015	423	Sociodemographics, Insurance Type, Geography

Study (Year)	Title	Study Design	Data Source	Study Years	Total Study n	Categories
	Reconstruction in a West Virginia Tertiary Care Hospital		(CAMC) cancer registry			
Iskandar (2015)	Factors influencing incidence and type of postmastectomy breast reconstruction in an urban multidisciplinary cancer center	Retrospective Cohort	Multi-institutional	2010-2011	258	Surgeon, Sociodemographics, Insurance Type, Hospital Type
Johnstone (2023)	Racial Disparities in Postoperative Breast Reconstruction Outcomes: A National Analysis	Retrospective Cohort	Multi-institutional	2003-2019	104,714	Sociodemographics
Kamali (2018)	Immediate Breast Reconstruction among Patients with Medicare and Private Insurance: A Matched Cohort Analysis	Retrospective Cohort		1992–2013	21,402	Sociodemographics, Insurance Type
Kamali (2017)	Differences in the Reporting of Racial and Socioeconomic Disparities among Three Large National Databases for Breast Reconstruction	Retrospective Cohort	SEER NSQIP NIS	2005-2012	607,867	Sociodemographics, Breast Cancer Type
Le Blanc (2022)	Association of Medicaid Expansion with Post-mastectomy Reconstruction Rates	Retrospective Cohort	National Cancer Database	2007-2017	1,103,973	Insurance Type, Geographic, Sociodemographics
Levine (2012)	A 10-year review of breast reconstruction in a university-based public hospital	Retrospective Cohort	Single hospital database	2001-2009	309	Sociodemographics
Li (2023)	Cultural Beliefs Regarding Breast Reconstruction in a Minority Group	Survey	Single Institution	2023	8	Decision-making, Communication
Liston (2022)	The State of Breast Cancer Reconstruction in Virginia: An Evidence-Based Framework for Identifying Locoregional Health Disparities	Retrospective Cohort	Virginia Department of Health Cancer Registry	2000-2018	78,682	Sociodemographics, Geography, Surgeon Density

Study (Year)	Title	Study Design	Data Source	Study Years	Total Study n	Categories
Llaneras (2023)	Post-Mastectomy Patients in an Urban Safety-Net Hospital: How Do Safety-Net Hospital Breast Reconstruction Rates Compare to National Breast Reconstruction Rates?	Retrospective Cohort	Single institution	2015-2019	259	Sociodemographics
Mahmoudi (2015)	Effect of federal and state policy changes on racial/ethnic variation in immediate postmastectomy breast reconstruction	Retrospective Cohort	New York State Inpatient Database	1998-2006	44,621	Sociodemographics, Insurance Type
Mahmoudi (2017)	Association of a Policy Mandating Physician-Patient Communication With Racial/Ethnic Disparities in Postmastectomy Breast Reconstruction	Retrospective Case-Controlled	Multi-institutional	2008-2011	42 346	Sociodemographics, Communication
Mandelbaum (2021)	National trends and predictors of mastectomy with immediate breast reconstruction	Retrospective Cohort	NIS	2005-2014	729,340	Sociodemographics, Income, Hospital Type
Matros (2010)	Sociodemographics, referral patterns, and Internet use for decision-making in microsurgical breast reconstruction	Survey	Single institution	2004-2007	377	Sociodemographics, Communication, Education
Meade (2022)	Breaking Barriers to Breast Reconstruction among Socioeconomically Disadvantaged Patients at a Large Safety-net Hospital	Retrospective Cohort	Single institution	2016-2019	645	Sociodemographics, Income, Insurance Type, Communication
Momoh (2019)	Patterns and Correlates of Knowledge, Communication, and Receipt of Breast Reconstruction in a Modern Population-Based Cohort of Patients with Breast Cancer	Retrospective Cohort	SEER	2013-2014	936	Sociodemographics, Income, Surgeon

Study (Year)	Title	Study Design	Data Source	Study Years	Total Study n	Categories
Moroni (2022)	Disparities in Access to Postmastectomy Breast Reconstruction: Does Living in a Specific ZIP Code Determine the Patient's Reconstructive Journey?	Retrospective Cohort	Single Institution	2014-2019	5,835	Sociodemographics, Income, Geography
Nassser (2023)	Regional Implicit Racial Bias and Rates of Breast Reconstruction, Complications, and Cost Among US Patients With Breast Cancer	Retrospective Cohort Implicit Bias Testing	NIS	2009-2019	52,115	Physician Implicit Bias
Nkana (2021)	Evaluation of Racial Disparities in Postoperative Outcomes Following Breast Reconstruction at a Single Institution in Wisconsin	Retrospective Cohort	Single institution NSQIP	2009-2020	1,140	Sociodemographics
Obeng-Gyasi (2022)	Is Medicaid Expansion Narrowing Gaps in Surgical Disparities for Low-Income Breast Cancer Patients?	Retrospective Case-controlled	National Cancer Database	2010-2017	6,131	Sociodemographics, Income, Insurance Type, Mandates
Obinero (2024)	We are moving the needle: Improving racial disparities in immediate breast reconstruction	Retrospective Cohort	NSQIP	2012-2021	119,043	Sociodemographics
Odom (2018)	A Cross-Sectional Study of Variations in Reimbursement for Breast Reconstruction: Is A Healthcare Disparity On the Horizon?	Retrospective Cohort Cross-Sectional	Single institution	2005-2015	2,691	Sociodemographics, Insurance Type, Reimbursement
Offodile (2015)	Racial disparities in the type of postmastectomy reconstruction chosen	Retrospective Cohort	NSQIP	2005-2011	44,597	Sociodemographics
Onega (2014)	The influence of race/ethnicity and place of service on breast reconstruction for Medicare	Retrospective Cohort	SEER	2005-2009	17,958	Sociodemographics, Insurance Type

Study (Year)	Title	Study Design	Data Source	Study Years	Total Study n	Categories
	beneficiaries with mastectomy					
Ramalingam (2021)	Improvement in Breast Reconstruction Disparities following Medicaid Expansion under the Affordable Care Act	Retrospective Cross-Sectional	NCDB	2010–2017	443,607	Sociodemographics, Insurance Type, Income, Education
Rosson (2008)	Multilevel analysis of the impact of community vs patient factors on access to immediate breast reconstruction following mastectomy in Maryland	Retrospective Cohort	Maryland Hospital Discharge Database	1995-2004	17,925	Sociodemographics, Education, Income, Geography
Schumacher (2020)	Utility of health services regions in examining socioeconomic disparities in receipt of breast reconstruction	Retrospective Cohort	Wisconsin Cancer Reporting System	2009-2014	6,349	Sociodemographics, Geography
Sergesketter (2019)	Decline in Racial Disparities in Postmastectomy Breast Reconstruction: A Surveillance, Epidemiology, and End Results Analysis from 1998 to 2014	Retrospective Cohort	SEER	1998-2014	346,418	Sociodemographics
Sharma (2016)	Race and Breast Cancer Reconstruction: Is There a Health Care Disparity?	Retrospective Cohort	Multiinstitutional	2000-2013	2,533	Sociodemographics, Income, Insurance Type
Shippee (2014)	Health insurance coverage and racial disparities in breast reconstruction after mastectomy	Retrospective Cohort	NIS	2002-2006	45,465	Sociodemographics, Insurance Type, Hospital Type
Siegel (2020)	Treatment at Academic Centers Increases Likelihood of Reconstruction After Mastectomy for Breast Cancer Patients	Retrospective Cohort	National Cancer Database	2004-2015	860,509	Hospital Type, Sociodemographics, Income, Insurance Type
Sisco (2012)	Have we expanded the equitable delivery of postmastectomy	Retrospective Cohort	NCDB	1998-2007	452,903	Sociodemographics, Income, Geography, Insurance Type

Study (Year)	Title	Study Design	Data Source	Study Years	Total Study n	Categories
	breast reconstruction in the new millennium? Evidence from the national cancer data base					
Stankowski (2023)	Factors Associated With Socioeconomic Disparities in Breast Reconstruction: Perspectives of Wisconsin Surgeons	Interview	Multi-institutional	2022	15	Surgeon, Education, Insurance Type
Stankowski (2022)	Barriers to breast reconstruction for socioeconomically disadvantaged women	Retrospective Cohort	Wisconsin Cancer Reporting System (WCRS)	2009-2014	1804	Sociodemographics, Insurance Type, Geography
Tseng (2004)	The effect of ethnicity on immediate reconstruction rates after mastectomy for breast cancer	Retrospective Cohort	Single institution	2001-2002	1004	Sociodemographics, Surgeon
Tseng (2010)	Sacramento area breast cancer epidemiology study: use of postmastectomy breast reconstruction along the rural-to-urban continuum	Retrospective Cohort	SEER	2000-2006	3552	Sociodemographics, Geography
Tung (2019)	Factors Leading to Decreased Rates of Immediate Postmastectomy Reconstruction	Retrospective Cohort	Multi-institutional	2006-2013	293	Insurance Type, Hospital Type, Sociodemographics
Vora (2018)	Reconstruction among patients undergoing mastectomy: the effect of surgical deserts	Retrospective Cohort	NIS	2007-2011	9325	Sociodemographics, Income, Insurance Type, Geography
Wang (2023)	Evaluating Disparities in Pathways to Breast Reconstruction	Retrospective Cohort	Single institution	2017-2018	218	Sociodemographics, Insurance Type, Geography
Weidman (2014)	Outcomes of patients in rural communities undergoing autologous breast reconstruction: A comparison of cost and patient demographics with implications for rural health policy	Retrospective Cohort	NIS	2012-22019	93,205	Hospital Type, Sociodemographics, Income, Insurance Type, Geography

Study (Year)	Title	Study Design	Data Source	Study Years	Total Study n	Categories
Wexelman (2014)	Socioeconomic and geographic differences in immediate reconstruction after mastectomy in the United States	Retrospective Cohort	NIS	2008	14,764	Sociodemographics, Income, Insurance Type, Geography
White (2023)	Disparities in post-mastectomy reconstruction use among American Indian and Alaska Native women	Retrospective Cohort	NCD	2004-2007	416,016	Sociodemographics
Wirth (2018)	Geographic variations in racial disparities in postmastectomy breast reconstruction: A SEER database analysis	Retrospective Cohort	SEER	2000-2013	182,396	Sociodemographics, Geography
Xie (2015)	Federal Health Coverage Mandates and Health Care Utilization: The Case of the Women's Health and Cancer Rights Act and Use of Breast Reconstruction Surgery	Retrospective Case-Controlled	SEER	1998-2000	15,737	Mandates
Yalamanchili (2021)	A look at racial and socioeconomic disparities in post-mastectomy breast reconstruction at a midwestern academic hospital	Retrospective Cohort	Multi-institutional	2013-2018	487	Sociodemographics, Income, Geography, Hospital Type
Yang (2013)	Trends in immediate breast reconstruction across insurance groups after enactment of breast cancer legislation	Retrospective Cohort	NIS	2000-2009	168,236	Mandates, Insurance Type
Yang (2018)	Hispanic Breast Cancer Patients Travel Further for Equitable Surgical Care at a Comprehensive Cancer Center	Retrospective Cohort	Stanford Cancer Registry Database	2010-2014	1765	Sociodemographics, Insurance Type
Yang (2013)	Racial disparities in immediate breast reconstruction after mastectomy: impact of	Retrospective Cohort	Pennsylvania Health Care Cost Containment	1994-2004	20,629	Mandates, Sociodemocraphics

Study (Year)	Title	Study Design	Data Source	Study Years	Total Study n	Categories
	state and federal health policy changes		Council Database			
Zaveri (2022)	Racial Disparities in Time to Treatment Persist in the Setting of a Comprehensive Breast Center	Retrospective Cohort	Single Institution	2012-2018	2,094	Sociodemographics, Hospital Type
